# CYX-5, a G-protein biassed MOP receptor agonist, DOP receptor antagonist and KOP receptor agonist, evokes constipation but not respiratory depression relative to morphine in rats

**DOI:** 10.1007/s43440-023-00446-8

**Published:** 2023-01-13

**Authors:** Mohammad Zafar Imam, Andy Kuo, Sussan Ghassabian, Yunxin Cai, Yajuan Qin, Tingyou Li, Maree T. Smith

**Affiliations:** 1 School of Biomedical Sciences, Faculty of Medicine, The University of Queensland, St Lucia Campus, Brisbane, QLD 4072 Australia; 2 School of Pharmacy, Nanjing Medical University, Nanjing, Jiangsu China

**Keywords:** CYX-5, Intracerebroventricular (*icv*), Warm water tail flick test, Gastrointestinal motility, Charcoal meal, Castor oil-induced diarrhoea, Whole body plethysmography, Hypercapnia, Minute ventilation

## Abstract

**Background:**

Strong opioid analgesics such as morphine alleviate moderate to severe acute nociceptive pain (e.g. post-surgical or post-trauma pain) as well as chronic cancer pain. However, they evoke many adverse effects and so there is an unmet need for opioid analgesics with improved tolerability. Recently, a prominent hypothesis has been that opioid-related adverse effects are mediated by β-arrestin2 recruitment at the µ-opioid (MOP) receptor and this stimulated research on discovery of G-protein biassed opioid analgesics. In other efforts, opioids with MOP agonist and δ-opioid (DOP) receptor antagonist profiles are promising for reducing side effects *c.f.* morphine. Herein, we report on the in vivo pharmacology of a novel opioid peptide (CYX-5) that is a G-protein biassed MOP receptor agonist, DOP receptor antagonist and kappa opioid (KOP) receptor agonist.

**Methods:**

Male Sprague–Dawley received intracerebroventricular bolus doses of CYX-5 (3, 10, 20 nmol), morphine (100 nmol) or vehicle, and antinociception (tail flick) was assessed relative to constipation (charcoal meal and castor oil-induced diarrhoea tests) and respiratory depression (whole body plethysmography).

**Results:**

CYX-5 evoked naloxone-sensitive, moderate antinociception, at the highest dose tested. Although CYX-5 did not inhibit gastrointestinal motility, it reduced stool output markedly in the castor oil-induced diarrhoea test. In contrast to morphine that evoked respiratory depression, CYX-5 increased tidal volume, thereby stimulating respiration.

**Conclusion:**

Despite its lack of recruitment of β-arrestin2 at MOP, DOP and KOP receptors, CYX-5 evoked constipation, implicating a mechanism other than β-arrestin2 recruitment at MOP, DOP and KOP receptors, mediating constipation evoked by CYX-5 and potentially other opioid ligands.

## Introduction

Historically, the pain-relieving and adverse effects evoked by strong opioid analgesics were thought to be mediated by the same intracellular mechanisms following µ-opioid (MOP) receptor activation [1–4]. This led to the belief that it would not be possible to separate the pain-relieving and adverse effects evoked by strong opioid analgesics. However, this notion was challenged by reports of increased antinociception and a reduction in some morphine-related adverse effects in β-arrestin2 knockout (KO) mice [5, 6]. These findings in β-arrestin2 KO mice, led to development of the hypothesis that the antinociceptive effects of MOP receptor agonists are mediated by G-protein coupled intracellular signalling pathways whereas recruitment of β-arrestin2 at the MOP receptor transduces opioid-related adverse effects [5, 6]. To test this hypothesis, a concerted effort in the past decade has been directed at discovery of novel G-protein biassed MOP receptor agonists including oliceridine (also called TRV130) and PZM21 [7]. Although intravenous oliceridine was approved by the US Food and Drug Administration in 2020 for use in hospitals when other treatment options are inadequate, it has a boxed warning on addiction and life-threatening respiratory depression [8]. This outcome has led to renewed research attention on other strategies for discovery of novel opioid analgesic ligands with superior adverse effect profiles relative to the ‘gold standard’ morphine.

One strategy for discovery of novel opioid analgesic ligands with improved preclinical therapeutic indices relative to morphine, is the multi-targeted opioid ligand strategy [9–11]. Examples include dual-targeted ligands that have both MOP and DOP receptor agonist activity [12], MOP/DOP receptor agonists that are also biassed ligands [12–14] as well as MOP receptor agonist/DOP receptor antagonist ligands [14, 15].

Interestingly, co-administration of a MOP receptor agonist with a DOP receptor antagonist in rodent pain models, evoked antinociception with improved tolerability, less analgesic tolerance and reduced abuse liability compared with a MOP receptor agonist alone [16]. This suggested that ligands with MOP receptor agonist and DOP receptor antagonist profiles may retain analgesic efficacy with reduced propensity to evoke opioid-related adverse effects [14–17]. In our previous work, [18] we described the discovery of CYX-6 (also called compound 7) and CYX-5 (also called compound 9) as more stable analogues of the endogenous opioid peptide, endomorphin-2 (EM-2) in the context of a structure–activity-study involving replacement of one or more amino acids with unnatural amino acids. In brief, the tetrapeptide, CYX-6 (H-Dmt-Pro-Tmp-Tmp-NH_2_ [18]; Dmt: 2,6-dimethyl-l-tyrosine; Tmp: 2,4,6-trimethyl-L-phenylalanine) was a G-protein biassed MOP receptor agonist, a DOP receptor antagonist and a KOP receptor antagonist in vitro [18]. In rats, single intracerebroventricular (*icv*) bolus doses of CYX-6 evoked potent, dose-dependent antinociception and it was devoid of constipation and respiratory depressant effects, in contrast to the constipating and respiratory depressant effects of *icv* bolus doses of the positive control, morphine [19].

**Fig. 1:**
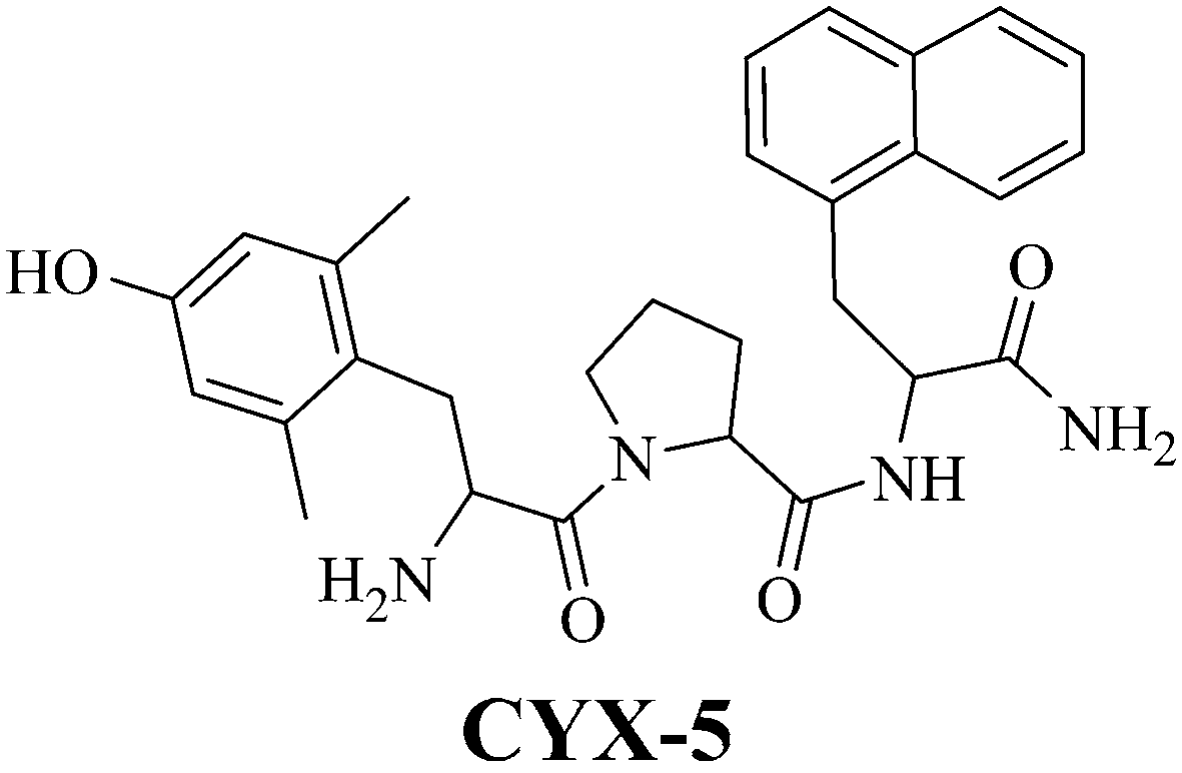
Chemical structure of CYX-5

By comparison, CYX-5 is a truncated analogue of EM-2 (H-Dmt-Pro-Nal-NH_2_; Nal: 3-(1-naphthyl)-l-alanine) (Fig. 1) [18]. In vitro, CYX-5, like CYX-6, was a G-protein biassed ligand at MOP, DOP and KOP receptors [18]. CYX-5 also bound with high affinity at the MOP receptor (*K*_i_ = 0.70 ± 0.08 nM (mean ± SEM)) but low affinity at the DOP receptor (*K*_i_ = 120.8 ± 10.2 nM) [18]. CYX-5 also demonstrated potent MOP receptor agonism ex vivo in the small intestinal myenteric plexus muscle preparation of the guinea-pig [18]. In the forskolin-stimulated cAMP assay in HEK-cells expressing murine MOP, DOP or KOP receptors, CYX-5 evoked MOP and KOP receptor agonism and DOP receptor antagonism [18]. Taken together, this in vitro and ex vivo pharmacological profile of CYX-5 raises the possibility that it may have a different in vivo profile to CYX-6 as CYX-6 has KOP receptor antagonist activity whilst CYX-5 has KOP receptor agonist activity.

Here we report on the antinociceptive, constipating and respiratory depressant effects evoked by single *icv* bolus doses of CYX-5 relative to morphine and vehicle, in male rats. The *icv* dosing route was chosen because direct administration of investigational agents into the cerebrospinal fluid of the lateral ventricle of the brain enables intrinsic efficacy and adverse effect profiles to be evaluated without the confounding effects of drug metabolism, due to the low capacity of the CNS to metabolise exogenous compounds [20]. Additionally, based on the hydrophilicity of CYX-5, it is unlikely to cross the blood–brain–barrier (BBB) and the *icv* dosing route bypasses BBB penetration. Furthermore, the *icv* dosing route avoids systemic pharmacokinetic effects that have the potential to confound data interpretation due to the likely poor ability of CYX-5 to cross the BBB and/or the formation of the neuro-excitatory metabolite of the positive control, morphine, morphine-3-glucuronide, in the liver of rodents [21].

## Materials and methods

### Chemicals

Professor Tingyou Li and colleagues synthesised CYX-5 using our published method [18]. Other chemicals/reagents were from commercial sources as follows: morphine hydrochloride (Royal Brisbane and Women’s Hospital pharmacy, Herston, Queensland, Australia), xylazine (Troy Laboratories Pty Ltd, Smithfield, New South Wales, Australia), zoletil (Virbac Australia Pty Limited, Milperra, New South Wales, Australia), castor oil (Sigma-Aldrich, Castle Hill, NSW, Australia), malachite green dye (BDH Chemicals Ltd, Poole, United Kingdom); water for injection BP (Pfizer, West Ryde, NSW, Australia).

### Dosing solutions

As CYX-5 is water soluble, *icv* dosing solutions were prepared in sterile water for injection as was morphine hydrochloride and the vehicle was sterile water for injection. In a preliminary study of the metabolic stability of CYX-5 using rat liver microsomes (unpublished), the half-life was 0.96 h which is much longer than that of the parent peptide, EM-2 (reviewed in Smith et al. [8]). The good metabolic stability of CYX-5 combined with the low capacity of the CNS to metabolise exogenous compounds [20], indicates that CYX-5 is suitably stable for *icv* dosing and data interpretation. The *icv* doses of CYX-5 were chosen to include the maximum tolerated dose (20 nmol) that was identified in a pilot study, and two lower doses (3 and 10 nmol).

### Animals

Approval for the experiments was from the Molecular Biosciences Animal Ethics Committee of The University of Queensland (Approval number: TETRAQ/069/15/ARC/BOEHRINGER and CIPDD/068/18) for the periods March 17 2015 to March 17 2018 and March 9 2018 to March 9 2021, respectively. In vivo experiments complied with the requirements of the Australian Code of Practice for the Care and Use of Animals for Scientific Purposes (8th Edition, 2013) [22] and with those of the Committee for Research and Ethical Issues of the International Association for the Study of Pain. Sprague–Dawley (SD) rats (male, albino) were from the Animal Resources Centre (Canning Vale, Western Australia, Australia) and they were housed in groups of two or three per cage with free access to food and water. The holding facility was maintained at a mean (± standard deviation) temperature of 23 (± 3) °C with relative humidity of 40–70% and a 12 h light/12 h dark cycle.

A brief description of the methods is given in the next several paragraphs and we have published the detailed methods elsewhere [19].

After acclimatisation, rats with the desired weight range (190–230 g), were anaesthetized to facilitate implantation of an *icv* guide cannula with a 9 mm cannula plug that remained in place except during injection of the test or control item. Rats were kept warm and monitored during post-surgical recovery and they were given a 5–7 days recovery period before *icv* dosing and experimentation.

Rats were allocated randomly to receive an *icv* bolus dose of CYX-5 at 3, 10 or 20 nmol, morphine at 100 nmol or vehicle. Dosing solutions were coded and randomized prior to administration for blinding of the investigator. Data were unblinded after completion of experimentation and data analyses. Experimentation was performed in the light phase (6:00 a.m. to 6:00 p.m.) by the same investigator (M.Z.I.).

### Test item administration

Rats received a single *icv* injection (10 μl) of CYX-5, morphine or vehicle under a brief period of light anaesthesia. After completion of experimentation, rats were again lightly anaesthetized for administration of an *icv* injection of malachite green dye to verify correct cannula placement. Data exclusion criteria were: (i) incomplete injection of the test item because of backflow of the injected solution or (ii) an inadequate distribution of dye in the ventricles.

### Warm-water tail flick test

The tail flick test was used to assess the extent and duration of antinociception evoked by bolus doses of *icv* CYX-5 (3, 10 and 20 nmol) relative to *icv* morphine (100 nmol) and vehicle [19, 23]. To assess a role for opioid receptors in mediating antinociception, rats were dosed with naloxone (10 mg/kg; *sc*) 10 min prior *icv* dosing with CYX-5 at the highest dose tolerated (20 nmol).

### Gastrointestinal (GI) motility assessed using the charcoal meal test

This test involved administration of a 1 mL oral dose of a 10% suspension of charcoal in 5% gum arabic in rats, to assess the effects of *icv* doses of CYX-5, morphine and vehicle on GI motility [19]. The charcoal meal was given 30 min after *icv* dosing of CYX-5, morphine or vehicle. One hour later, the rats were euthanised and the intestine was collected for measurement of the following: (i) the distance from the pylorus to the front edge of the charcoal meal; (ii) the distance between the pylorus and the distal edge of the ileum. The intestinal transit index was determined by dividing measurement (i) by measurement (ii) [19].

### Opioid induced inhibition of diarrhoea induced by castor oil

After an overnight fast (16 h), the pre-dosing stool consistency of individual rats was recorded. This was followed by an *icv* dose of CYX-5, morphine or vehicle followed by an oral dose of castor oil (2.5 ml) at 5-min post-*icv* dosing by the blinded investigator. The weight and consistency of faeces were recorded pre-dose and hourly for 8 h post-dosing for individual animals. The scoring criteria for faeces consistency were as follows: 0 = normal faeces, 1 = well-shaped wet faeces, 2 = shapeless faeces, 3 = shapeless faeces with large amounts of liquid [19, 23]. A score of ≥ 2 for both weight and consistency was defined as diarrhoea [19, 23].

### Respiratory function using whole body plethysmography

The effects of *icv* CYX-5, morphine and vehicle on respiratory parameters in awake, freely moving rats were measured using a Buxco small animal whole body plethysmography (WBP) system (Buxco Electronics, Troy, New York, USA) as described previously by our group [19, 23]. After acclimatisation for 30–60 min, respiratory parameters were recorded for 45 min pre-dosing (three 15 min cycles) and for 2 h post-dosing (eight 15 min cycles). In each cycle, rats breathed normal air for 1 min, followed by a hypercapnic gas mixture (8% CO_2_, 21% O_2_ and 71% N_2_) for 5 min and then a 9 min recovery period breathing normal air [19, 23]. Respiratory parameters recorded by the blinded tester (M.Z.I.) included minute ventilation (MV), respiratory frequency (RF) and tidal volume (Tv) using the BioSystem XA software [19, 23]. The baseline was calculated as the mean difference in MV (ΔMV), RF (ΔRF) and Tv (ΔTv) during the pre-dosing exposure to normal air and the hypercapnic gas mixture [19, 23]. Following *icv* dosing with CYX-5, morphine or vehicle in individual animals, ΔMV, ΔRF and ΔTv were calculated for each cycle of the 2 h post-dosing period [19, 23].

### Statistical analyses

**Fig. 2:**
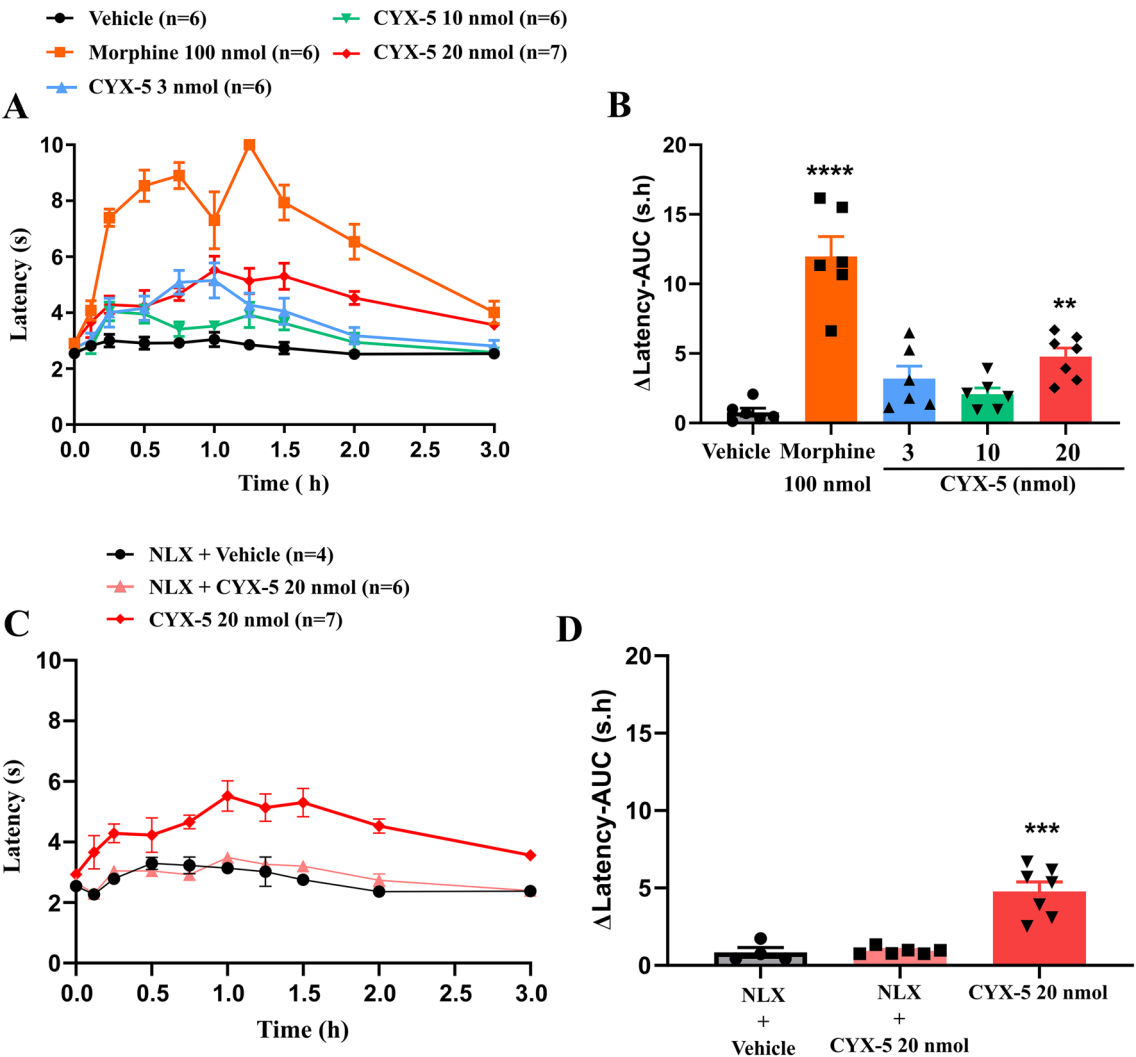
*Icv* CYX-5 evoked naloxone-sensitive antinociception (tail flick) in rats. **A** Mean (± SEM) tail flick latency versus time curves for *icv* CYX-5 (3 (*n* = 6), 10 (*n* = 6) and 20 nmol (*n* = 7)) relative to *icv* bolus doses of the positive control, morphine (100 nmol, *n* = 6) and the negative control, vehicle (*n* = 6). **B** Mean (± SEM) area under the increase in tail flick latency above baseline (ΔLatency) versus time curves for *icv* CYX-5 relative to morphine and vehicle. **C** Pre-treatment of rats with the opioid antagonist naloxone (10 mg/kg *sc*) at 10 min prior to *icv* CYX-5 (20 nmol), abolished CYX-5 antinociception. **D** In naloxone pre-treated rats, the extent and duration of *icv* CYX-5 antinociception quantified as the mean (± SEM) area under the ΔLatency versus time curve did not differ significantly from that for naloxone pre-treated rats administered *icv* vehicle (one-way ANOVA; **p < 0.01, ***p < 0.001, ****p < 0.0001)). The *icv* morphine and *icv* vehicle data from Eur J Pharmacol 2020;871:172918, Fig. 2, **A** and **B**, have been re-used with permission. Note that the CYX-5 data were generated concurrently in a blinded manner [19]

**Fig. 3:**
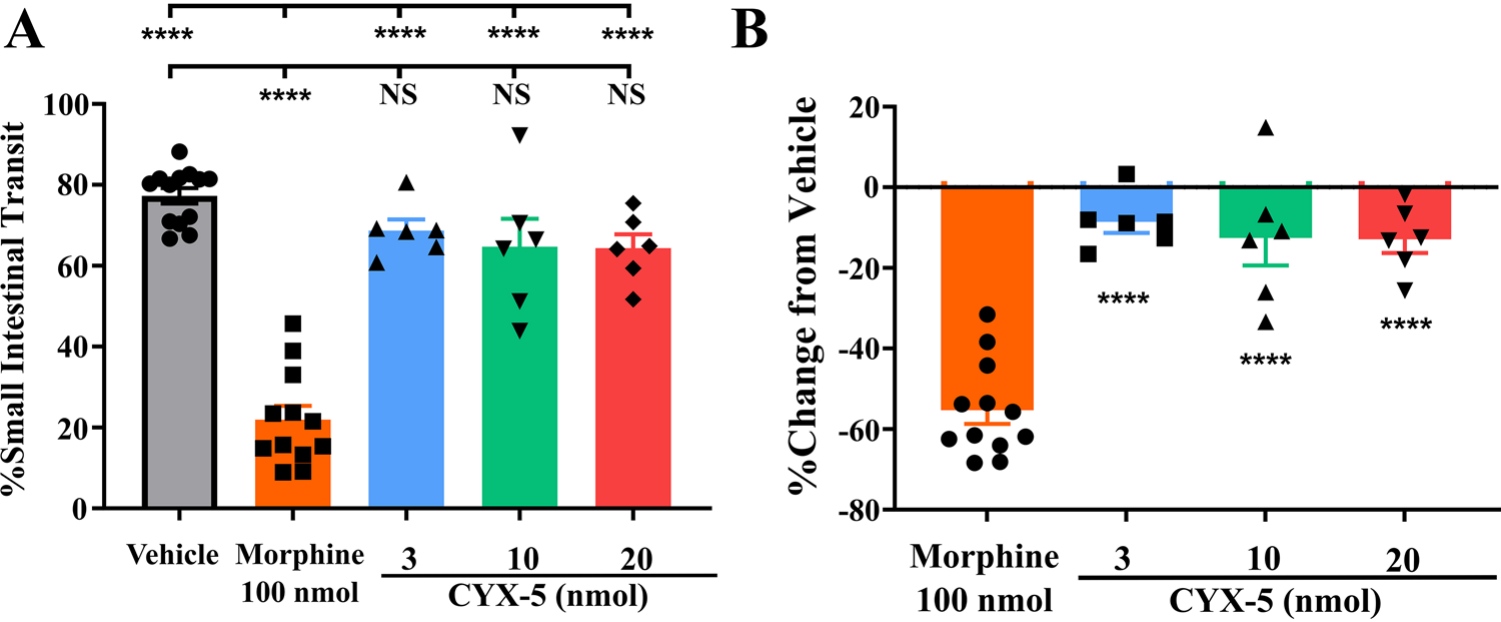
In rats, *icv* CYX-5 did not significantly alter intestinal motility in a manner similar to *icv* vehicle, and in contrast to *icv* morphine. **A** The intestinal transit of a charcoal meal given by gavage at 1 h prior to *icv* dosing in rats, did not differ significantly between animals given *icv* bolus doses of CYX-5 (3–20 nmol; *n* = 6/dose) or vehicle (*n* = 13) (one-way ANOVA, ****p < 0.0001). As expected, the positive control, *icv* morphine (100 nmol; *n* = 12), significantly impaired intestinal transit relative to *icv* vehicle (one-way ANOVA, ****p < 0.0001). **B** Additionally, the % change in intestinal transit evoked by *icv* CYX-5 did not differ significantly from that evoked by *icv* saline in contrast to the significant reduction evoked by *icv* morphine in rats (one-way ANOVA, ****p < 0.0001). The *icv* morphine and *icv* vehicle data from Fig. 3 of Eur J Pharmacol 2020;871:172,918, have been re-used with permission. Note that the CYX-5 data were generated concurrently in a blinded manner [19]

**Fig. 4:**
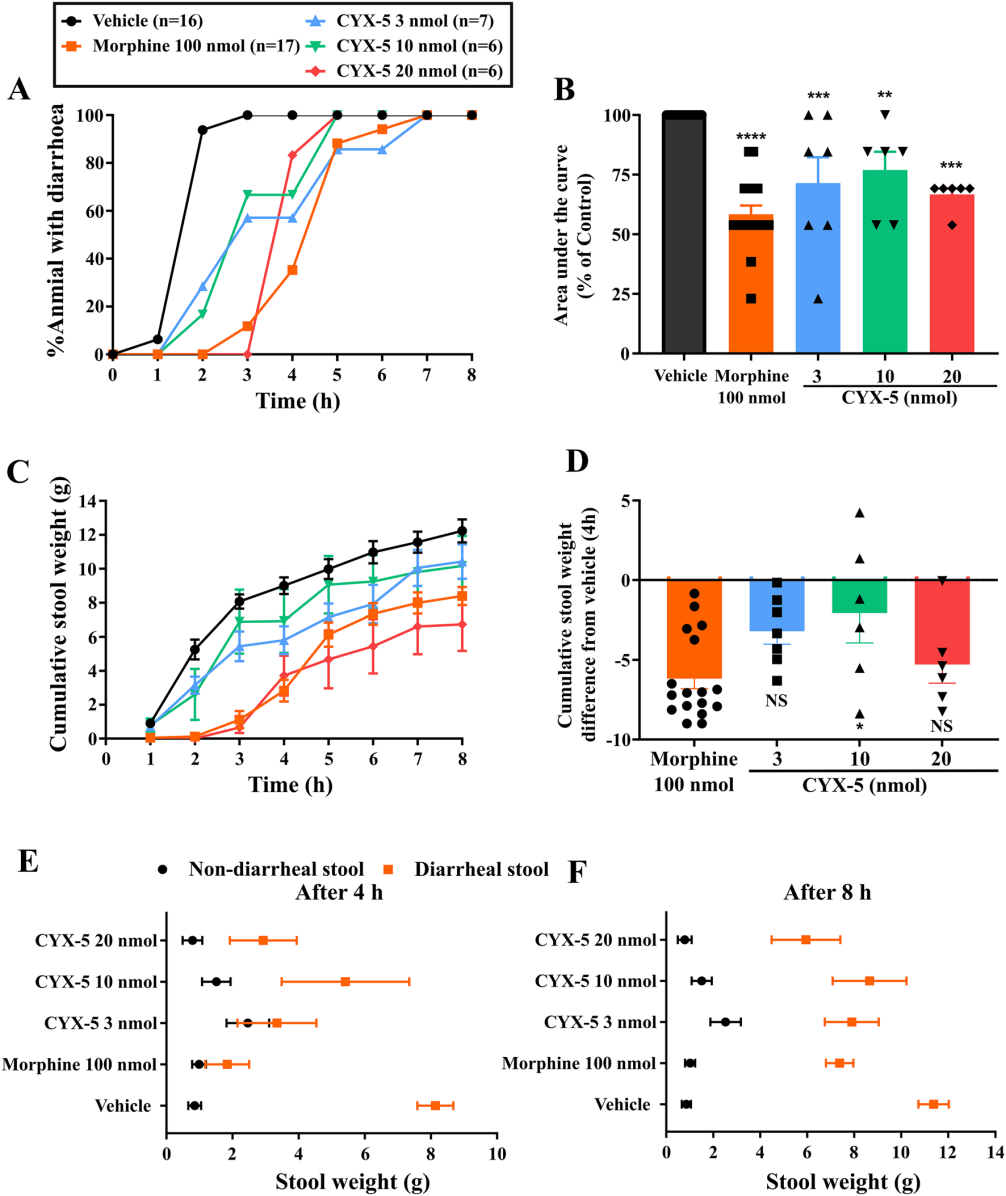
*Icv* CYX-5 inhibited castor oil-induced diarrhoea (3 nmol, *n* = 7; 10 nmol, *n* = 6; 20 nmol, *n* = 6), as a measure of constipation, in a manner similar to *icv* morphine (*n* = 17). **A** % animals with diarrhoea after an oral dose of castor oil over an 8 h post-dosing period. **B** Area under the curve (AUC) of % rats with diarrhoea (% of vehicle control, one-way ANOVA, **p < 0.01, ***p < 0.001, ****p < 0.0001). **C** Cumulative stool output after oral administration of castor oil. **D** Cumulative stool weight in the first 4 h post-dose period was significantly reduced in rats given either *icv* CYX-5 or *icv* morphine, relative to that for rats given *icv* vehicle (one-way ANOVA, *p < 0.05, *NS* not significant). **E**, **F** Comparison of the non-diarrhoeal and diarrhoeal stool consistency observed during the first 4 h (**E**) and at 8 h (**F**) post-dosing. The *icv* morphine and *icv* vehicle data from Fig. 4, panels **A**–**F**, of Eur J Pharmacol 2020;871:172,918, have been re-used with permission. Note that the CYX-5 data were generated concurrently in a blinded manner [19]

**Fig. 5:**
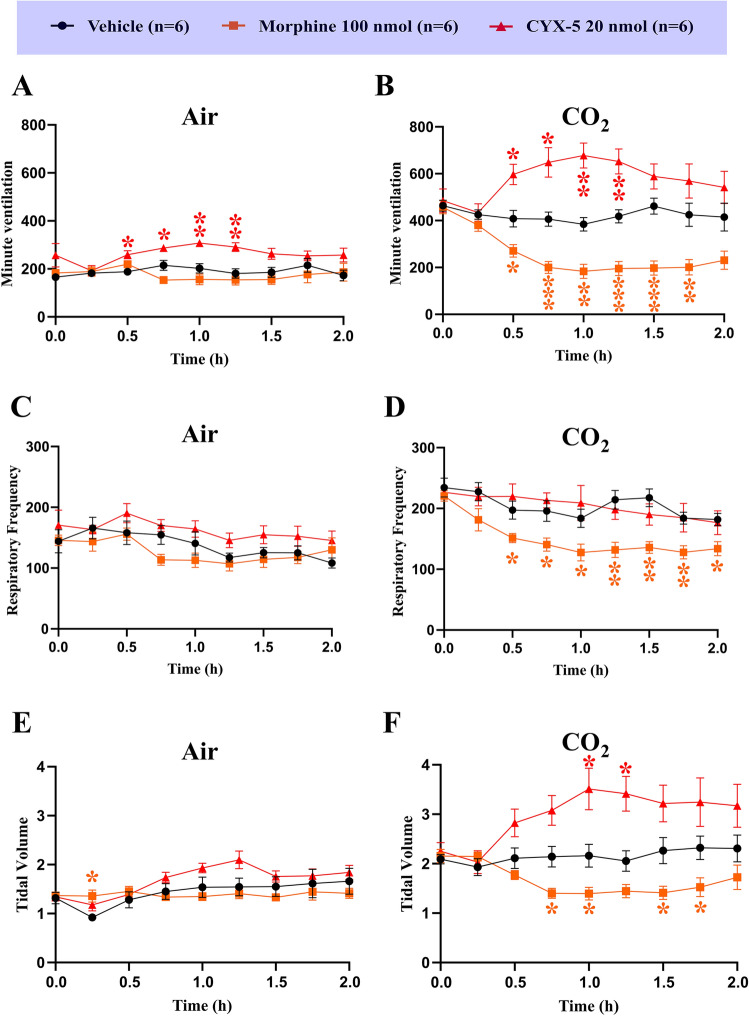
*Icv* CYX-5 stimulated respiratory function in rats. *Icv* CYX-5 at the highest tolerated dose (20 nmol (*n* = 6) did not depress minute ventilation or respiratory frequency significantly relative to vehicle (*n* = 6), for rats breathing either room air (**A**, **E**) or a hypercapnic gas mixture (**B**, **F**). By contrast, *icv* morphine at 100 nmol (*n* = 6) reduced minute ventilation at the time of peak effect (**B**). Morphine (100 nmol *icv*) but not CYX-5, reduced respiratory frequency significantly relative to *icv* vehicle for animals breathing the hypercapnic gas mixture (**D**). Tidal volume for rats given CYX-5 (20 nmol *icv*) was increased significantly relative to that for rats given *icv* vehicle and morphine irrespective of whether or not animals were breathing room air (**E**) or the hypercapnic gas mixture (**F**) (2-way ANOVA with a Dunnett’s post hoc multiple comparison test; *p < 0.05. **p < 0.01). The *icv* morphine and *icv* vehicle data from Fig. 5, panels **A**–**F**, of Eur J Pharmacol 2020;871:172,918, have been re-used with permission. Note that the CYX-5 data were generated concurrently in a blinded manner [19]

**Fig. 6:**
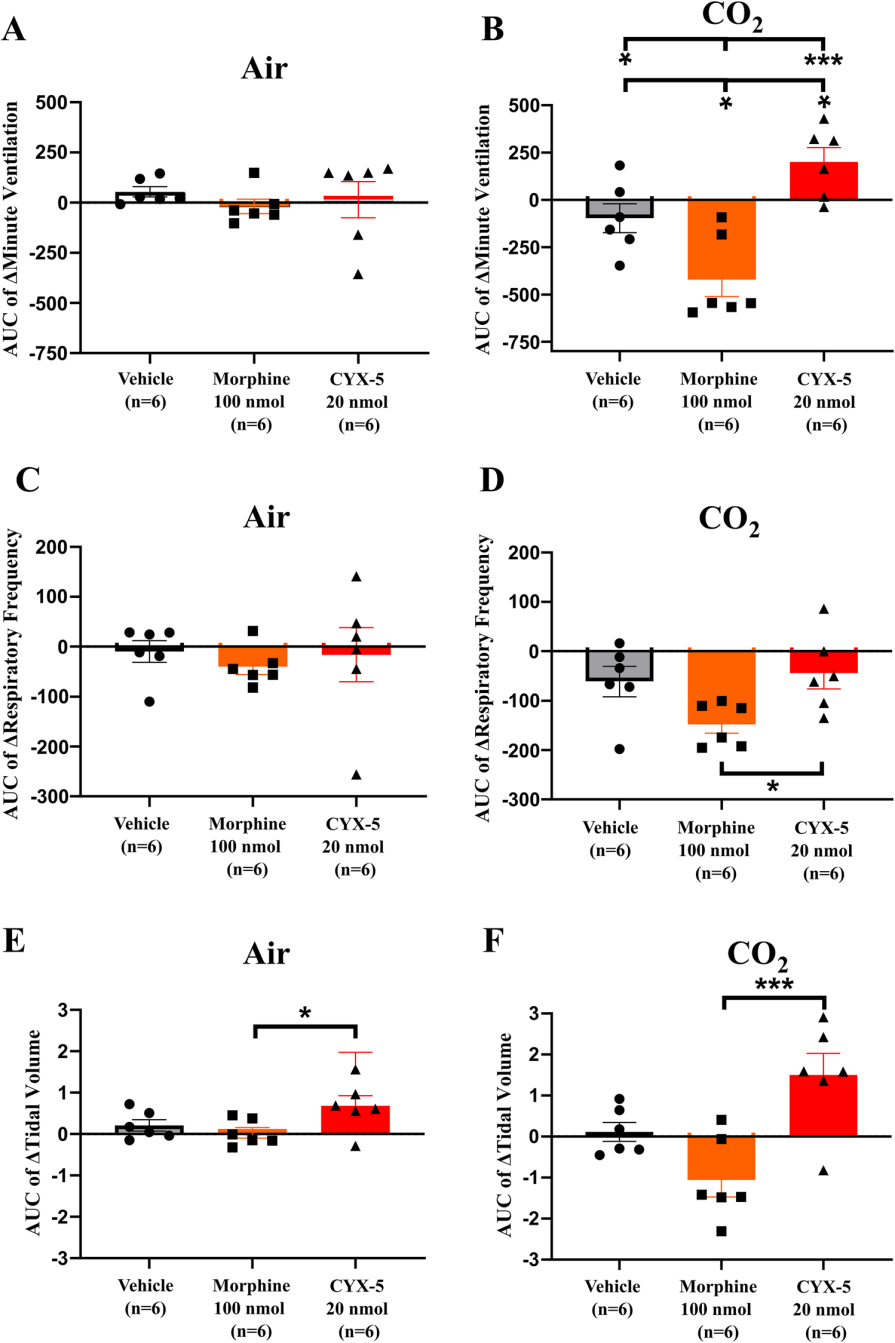
Effects of *icv* CYX-5 on the extent and duration of the change (change in area under the curve, ΔAUC) in minute ventilation, respiratory frequency and tidal volume in rats breathing room air or a hypercapnic gas mixture (**A**–**F**). *Icv* CYX-5 at 20 nmol (*n* = 6) increased the Δ minute ventilation AUC but it did not affect the Δ respiratory frequency AUC under hypercapnic conditions relative to the corresponding parameters for vehicle (**B**, **D**). By contrast, *icv* morphine (*n* = 6), reduced the Δ minute ventilation AUC relative to that for *icv* vehicle (*n* = 6). Interestingly, CYX-5 increased the Δ tidal volume AUC for rats breathing either room air (**E**) or the hypercapnic gas mixture (**F**) whereas morphine reduced the Δ tidal volume AUC relative to that for vehicle, but these did not reach statistical significance. However, for CYX-5, Δ minute ventilation AUC, Δ respiratory frequency AUC and Δ tidal volume AUC of CYX-5 all differed significantly from that for morphine for rats breathing the hypercapnic gas mixture (**B**, **D**, **F**) (one-way ANOVA. *p < 0.05, **p < 0.01, ***p < 0.001). The *icv* morphine and *icv* vehicle data from Fig. 6, panels **A**–**F**, of Eur J Pharmacol 2020;871:172,918, have been re-used with permission. Note that the CYX-5 data were generated concurrently in a blinded manner [19]

Data are presented as mean ± standard error of mean (SEM). One-way analysis of variance was used for analysing the following data: ΔLatency-AUC of warm water tail flick data (Fig. 2B, D); % Small intestinal transit of charcoal meal data and % change of transit relative to vehicle (Fig. 3A, B); AUC of % rats with diarrhoea (% of vehicle control) (Fig. 4B), Cumulative stool weight difference from vehicle (4 h) (Fig. 4D); ΔMV-AUC, ΔRF-AUC and ΔTv-AUC (Fig. 6A–F). Two-way analysis of variance was used for the following data: MV, RF and Tv data for 2 h WPB test (Fig. 5A–F). The analyses were implemented in GraphPad Prism v9.0.0 (GraphPad Software, Inc., La Jolla, CA USA). Dunnett's multiple comparison test was used for post hoc analysis of all the data. For individual animals in the tail flick test, the increase in tail flick latency above baseline (ΔLatency) was calculated for every assessment time point, and the areas under the ΔLatency versus time curve (AUC) values were estimated using trapezoidal integration as using GraphPad™ Prism v9.0.0. For the castor oil-induced diarrhoea test, the percentage of rats with diarrhoea (%AD) was determined for each time point and plotted against time for individual animals. The areas under the curve (%AD-AUC) values were calculated using GraphPad™ Prism v9.0.0. A value of *p* ≤ 0.05 was the statistical significance criterion. For one-way ANOVA, *F* values are expressed as *F*_df of treatment, residual_. For two-way ANOVA, *F* values are expressed as *F*_df of treatment, time, interaction/residual_.

## Results

### CYX-5 evokes moderate antinociception relative to morphine

For rats with a baseline tail flick latency in the range 2.0–3.5 s to a warm-water stimulus, single bolus doses of *icv* CYX-5 evoked moderate antinociception at the highest dose (20 nmol) tolerated (Fig. 2A). By comparison, single *icv* bolus doses of the positive control, morphine (100 nmol), evoked pronounced antinociception characterised by a biphasic profile similar to previous work from our laboratory [23] (Fig. 2A). For *icv* bolus dose of CYX-5 at 3, 10 and 20 nmol, the mean (± SEM) peak tail flick latencies were 5.2 (± 0.6), 4.0 (± 0.3) and 5.5 (± 0.5) s respectively and the mean time to peak effect was 1 h post-dose (Fig. 2A). By comparison, the first and second antinociceptive peak latencies for morphine were 8.9 (± 0.5) and 10.0 (± 0.0) s respectively and the corresponding mean times to peak effect were 0.75 and 1.5 h, respectively (Fig. 2A). A preliminary experiment involving *icv* administration of CYX-5 at 30 nmol, resulted in pronounced neuro-excitatory side effects (including convulsions, barrel roll, wet-dog shakes, excessive alertness, etc.), so this and higher doses were not assessed. Clinical signs of neuroexcitation were mild, intermittent, and transient in ~ 50% of rats dosed with *icv* CYX-5 at 20 nmol. CYX-5 at 20 nmol also induced postural abnormalities including a flattened torso, splayed legs and impaired balance (ataxia) for up to 90 min.

The mean (± SEM) ΔLatency-AUC values for CYX-5 at 3, 10 and 20 nmol doses were 3.2 (± 0.9) s.h, 2.1 (± 0.5) s.h and 4.8 (± 0.6) s.h, respectively (Fig. 2B). The corresponding values for morphine and vehicle were 12.0 (± 1.4) s.h and 0.8 (± 0.3) s.h respectively (Fig. 2B). The ΔLatency-AUC values for the 20 nmol dose of CYX-5 and morphine at 100 nmol were significantly different from the corresponding response evoked by an *icv* dose of the vehicle (*F*_4, 26_ = 27.48, *p* < 0.0001, one-way ANOVA). Naloxone pre-treatment (10 mg/kg, *sc*) completely attenuated antinociception evoked by *icv* CYX-5 at 20 nmol, confirming the antinociception evoked by CYX-5 was opioid receptor-mediated (Fig. 2C, D) (*F*_2, 14_ = 25.74, *p* < 0.0001, one-way ANOVA).

### Supraspinal CYX-5 has minimal effect on gut motility in rats

When assessed using the charcoal meal test, *icv* CYX-5 had a better gastrointestinal motility profile compared with *icv* administration of the positive control, morphine at 100 nmol (Fig. 3). For *icv* CYX-5, the mean (± SEM) percentage (%) of the small intestinal transit (%SIT) values for the 3, 10 and 20 nmol doses were 68.7 (± 2.7), 64.8 (± 6.8) and 64.4 (± 3.4) %, respectively (Fig. 3A). For the *icv* morphine (100 nmol) and vehicle-treated rats, the %SIT value was 22.0 (± 3.4) and 77.3 (± 1.9) % respectively (Fig. 3A, B). The %SIT values for CYX-5 doses were significantly different from that for morphine (*F*_4, 38_ = 50.51, *p* < 0.0001, one-way ANOVA) but not significantly different compared with that for vehicle (*p* > 0.05) (Fig. 3A).

Moreover, the % change in SIT relative to vehicle for the various CYX-5 doses were significantly different relative to that of morphine (100 nmol) (*F*_3, 26_ = 34.62, *p* < 0.0001, one-way ANOVA) (Fig. 3B). The results demonstrate the minimal effect of CYX-5 on GI motility.

### CYX-5 inhibits castor oil-induced diarrhoea as a measure of constipation

After *icv* administration of CYX-5 (3, 10 and 20 nmol), it took 5 h to develop diarrhoea in all animals (Fig. 4A). In comparison, the morphine and vehicle-treated animals developed diarrhoea by 7 and 3 h, respectively (Fig. 4A). The mean %AUC (± SEM) value for morphine was 58.4 (± 3.7) % and the corresponding values for CYX-5 at 3, 10 and 20 nmol were 72.6 (± 10.8), 78.5 (± 7.6) and 68.4 (± 2.6) %AD.h respectively (Fig. 4B). The mean %AUC (± SEM) values for all doses of CYX-5 (*p* < 0.01) and morphine (*p* < 0.0001) were significantly different from that for the vehicle (set at 100%) (*F*_4, 47_ = 16.84, *p* < 0.0001, one-way ANOVA).

CYX-5 at the 3 and 10 nmol doses, demonstrated less inhibition of castor oil-induced stool output during the first 4 h of the experiment compared with the morphine-treated animals (Fig. 4C). However, both CYX-5 (20 nmol) and morphine (100 nmol) delayed the onset of diarrhoea and significantly reduced the amount of stool output (Fig. 4C, D). The cumulative stool weight difference between CYX-5 and vehicle was significantly different from that for morphine only for the 10 nmol dose of CYX-5 at 4 h (*F*_3, 32_ = 3.57, *p* = 0.0247, one-way ANOVA) (Fig. 4D).

The cumulative stool weight-area under the curve (CSW-AUC) values for CYX-5 at the 3, 10 and 20 nmol doses were 11.7 (± 1.5), 13.4 (± 4.1) and 2.5 (± 0.9) g.h respectively at 4 h (*F*_4, 47_ = 26.43, *p* < 0.0001, one-way ANOVA). The CSW-AUC of morphine (100 nmol) and vehicle was 2.7 (± 0.8) g.h and 18.3 (± 1.1) g.h, respectively (*F*_4, 47_ = 26.43, *p* < 0.0001, one-way ANOVA).

Additionally, the total amount of diarrhoeal stool compared with non-diarrhoeal stool and the period of time when the stool of varying consistency was discharged by rats following *icv* administration of CYX-5, gave a deeper insight. Figure 4E, F display the diarrhoeal and non-diarrhoeal stools discharged by 4 h and 8 h after castor oil gavage. After 4 h, CYX-5 at 3 nmol produced a cumulatively large amount of stool discharge with a consistency from normal to diarrhoeal in a manner similar to vehicle. Although the amount of diarrhoeal stool discharge was lower in rats given *icv* doses at 10 and 20 nmol after 4 h, the amount of non-diarhoeal stool was higher than that for the morphine-treated animals (Fig. 4E).

Together, these findings show that *icv* CYX-5 at 20 nmol evoked constipation without significantly inhibiting GI motility in contrast to the positive control, *icv* morphine at 100 nmol, that inhibited both GI motility and castor oil-induced diarrhoea.

### Effect of CYX-5 on respiratory parameters in rats

When whole body plethysmography was used to assess the respiratory depressant effects of *icv* CYX-5 at the maximum tolerated dose (20 nmol) in awake, freely moving rats, there was a significant stimulatory effect on minute ventilation under hypercapnic conditions (*F*_2, 8, 15/120_ = 27.18, 1.364 and 8.789, *p* < 0.0001, = 0.2656 and < 0.0001; two-way ANOVA with a Dunnett’s post hoc multiple comparison test). The minute ventilation of the CYX-5 treated animals also differed significantly whilst breathing air at the 0.5 to 1.25 h time points (Fig. 5A) (*F*_2, 8, 15/120_ = 9.662, 1.284 and 6.797, *p* = 0.0020, = 0.2862 and < 0.0001; 2-way ANOVA with a Dunnett’s post hoc multiple comparison test). The effect on respiratory frequency was insignificant in rats that received *icv* CYX-5 (20 nmol) relative to rats administered morphine (100 nmol) during both normal air (*F*_2, 8, 15/120_ = 4.271, 4.745 and 4.681, *p* = 0.0340, = 0.0017 and < 0.0001; two-way ANOVA with a Dunnett’s post hoc multiple comparison test) and hypercapnic gas exposure (Fig. 5A–D) (*F*_2, 8, 15/120_ = 6.438, 13.42 and 14.51, *p* = 0.0096, < 0.0001 and < 0.0001; two-way ANOVA with a Dunnett’s post hoc multiple comparison test). Rather, a significant increase in the tidal volume was observed in CYX-5 treated rats that were breathing the hypercapnic gas mixture (Fig. 5F) (*F*_2, 8, 15/120_ = 10.08, 1.677 and 16.45, *p* = 0.0017, = 0.1974 and < 0.0001; two-way ANOVA with a Dunnett’s post hoc multiple comparison test).

The AUC values of Δ tidal volume differed from that for the vehicle but it was not significant (Fig. 6). However, Δ minute ventilation AUC (*F*_2, 15_ = 14.66, *p* = 0.0003), Δ respiratory frequency AUC (*F*_2, 15_ = 4.054, *p* = 0.0391; one-way ANOVA) and Δ tidal volume AUC of CYX-5 (*F*_2, 15_ = 9.834, *p* = 0.0019, one-way ANOVA) were all significantly different compared with morphine (*p* < 0.05) for rats exposed to the hypercapnic gas mixture (Fig. 5B, D, F).

## Discussion

Herein, we show in rats that single *icv* bolus doses of the EM-2 analogue, CYX-5, a G-protein biassed MOP/KOP receptor agonist and a DOP receptor antagonist [18] (Table 1), evoked moderate, naloxone-sensitive, antinociception at the highest dose tolerated (20 nmol) (Table 1). In our previous work [19], we reported that another EM-2 analogue, CYX-6, that is a G-protein biassed MOP receptor agonist and a DOP/KOP receptor antagonist [18] (Table 1) also evoked antinociception albeit more potently than CYX-5 herein [19]. For both CYX-5 and CYX-6 [19], doses could not be further escalated due to prominent neuro-excitatory side effects evoked by *icv* doses larger than 20 nmol. These neuro-excitatory behaviours may be mediated, at least in part, by a mechanism in common with that of dynorphin A (2–17), a major metabolite of the endogenous KOP receptor agonist, dynorphin A (1–17) [24]. Another possibility is indirect activation of the *N*-methyl-d-Aspartate receptor in a manner similar to the neuroexcitatory effects of the major morphine metabolite, morphine-3-glucuronide that accounts for > 50% of each oral morphine dose [25].

**Table 1:** Comparison of the in vitro activity at MOP, DOP and KOP receptors and in vivo efficacy (antinociception, constipation and respiratory function) for *icv* CYX-5 relative to *icv* CYX-6 and *icv* morphine in Sprague–Dawley rats

Opioid ligand	In vitro agonist or antagonist activity*			Antinociception**	Constipation**		Respiratory function**#	
	MOP receptor	DOP receptor	KOP receptor	Warm water tail flick test	Inhibition of GI motility	Inhibition of castor oil-induced diarrhoea	Depression of minute ventilation	Stimulation of minute ventilation
CYX-5	Agonist^a^	Antagonist^a^	Agonist^a^	√	√	X	X	√
CYX-6	Agonist^a^	Antagonist^a^	Antagonist^a^	√^b^	X^b^	X^b^	X^b^	√^b^
Morphine	Agonist^c^	Agonist^c^	Agonist^c^	√^b,d^	√^b,d^	√^b,d^	√^b,d^	X^b,d^

*In vitro inhibition of forskolin-stimulated cAMP formation using human embryonic kidney cells stably expressing MOP, DOP or KOP receptors

**icv dosing route in male Sprague–Dawley rats

^#^Assessed using whole body plethysmography under hypercapnic conditions in awake, freely moving rats

^a^Cai et al. [18]; ^b^Imam et al. [19]; ^c^Kuo et al. [36]; ^d^Kuo et al. [23]

Regarding constipation (Table 1*), icv* CYX-5 at 20 nmol did not inhibit GI motility, but it did attenuate castor oil-induced diarrhoea mimicking *icv* morphine (100 nmol) in this regard (Table 1). However, in our previous work [19], we reported that CYX-6 did not attuenuate castor oil-induced diarrhoea after *icv* dosing at 20 nmol in rats (Table 1) [19].A plausible explanation for these differences is that CYX-5 is a KOP receptor agonist (Table 1) whereas CYX-6 is a KOP receptor antagonist with both EM-2 analogues having MOP receptor agonist/DOP receptor antagonist activity (Table 1) [18]. In future work beyond the scope of that herein, MOP, DOP and KOP selective opioid antagonists and MOP, DOP and KOP receptor knockout mice will be used to gain additonal insight. In previous work by others, a MOP receptor agonist/DOP receptor antagonist had a lesser effect on GI transit and faecal output relative to that evoked by the peripherally selective MOP agonist, loperamide, following oral administration in mice [26]. In other work, *sc* 14-*O*-phenylpropyloxy-morphone, a highly potent, mixed MOP/DOP/KOP receptor agonist, had a favourable GI transit profile relative to morphine in mice. Specifically, 14-*O*-phenylpropyloxymorphone was 2.4-fold less potent for inhibition of GI transit compared with antinociception whereas *sc* morphine was 1.8-fold more potent for GI transit inhibition than for antinociception [27]. In other work, biphalin, an octapeptide analogue of enkephalin and a mixed MOP/DOP receptor agonist, at 5 mg/kg *ip*, decreased intestinal transit, colonic propulsion and delayed initiation of castor oil-induced diarrhoea [28]. Taken together, our findings and those of others highlight the potential importance of differential opioid receptor activity profiles (agonist/antagonist) at each of MOP, DOP and KOP receptors with regard to the evoked constipation profile in rats.

As both CYX-5 and CYX-6 were G-protein biassed opioid ligands, the inhibitory effect of *icv* CYX-5 on stool hydration in the castor oil-induced diarrhoea test, was somewhat unexpected. Our findings on the inhibitory effects of CYX-5 on castor oil-induced diarrhoea suggest that β-arrestin2 does not have a major role in mediating this dimension of constipation, at least in rats. This notion is supported by previous work by others whereby the constipation profiles of the biassed MOP receptor agonists, oliceridine (TRV130) and PZM21, in rodents [29, 30] were less impressive than expected based upon extrapolation of earlier findings for morphine in MOP receptor-KO mice [31]. Specifically in mice, *sc* doses of the G-protein biassed MOP receptor agonist, oliceridine, evoked robust warm water tail flick antinociception that appeared to be resistant to tolerance with repeated administration [29]. However, in work by others, antinociceptive tolerance did develop in mice administered PZM21 at 40 mg/kg *sc* for 3 consecutive days [32]. Regarding constipation, oliceridine inhibited faecal output and colonic propulsion markedly, without development of tolerance to these effects after repeated administration [29], which is undesirable. PZM21, another biassed MOP receptor agonist that evoked promising antinociception (40 mg/kg *sc*), also reduced faecal output significantly as an indicator of constipation, at a dose (20 mg/kg *sc*) that was lower than the antinociceptive dose [30]. This latter effect is similar to morphine that was 1.8-fold more potent for GI transit inhibition than for evoking antinociception in mice [27].

Regarding impact on respiratory function herein, single *icv* doses of CYX-5 at the highest dose tolerated (20 nmol), increased minute ventilation in awake, freely moving rats exposed to 5-min epochs of a hypercapnic gas mixture in contrast to the respiratory depressant effects of the positive control, *icv* morphine (100 nmol) (Table 1). This effect of *icv* CYX-5 in rats was underpinned by an increase in tidal volume but without a change in respiratory frequency. By comparison in our previous work, *icv* doses of CYX-6 at 20 nmol did not depress minute ventilation significantly under hypercapnic conditions in contrast to respiratory depression evoked by *icv* morphine (100 nmol) in awake freely moving rats (Table 1) [19]. Although CYX-6 like morphine, reduced respiratory frequency relative to vehicle, CYX-6 increased tidal volume relative to morphine and vehicle in these animals [19]. These differential effects of *icv* CYX-5 and CYX-6 on parameters of respiratory function in rats under hypercapnic conditions, despite both EM-2 analogues being G-protein biassed ligands at MOP, DOP and KOP receptors as well as MOP agonists and DOP antagonists, suggest that these differences may be underpinned by the KOP receptor agonist activity of CYX-5 in contrast to the KOP receptor antagonist activity of CYX-6 [18].

Close inspection of the effects of *icv* CYX-5 on respiratory parameters in rats breathing room air vis-à-vis 5 min epochs of a hypercapnic gas mixture (8% CO_2_ in air), highlight the importance of the latter clinically relevant condition, to unmask the effects of CYX-5 and morphine respectively, on parameters of respiration (Figs. 5, 6). Additionally, comparison of the respiratory stimulant effects of the biassed MOP/KOP agonist-KOP antagonist, CYX-5, with those for the biassed MOP agonist, PZM21 (40 mg/kg *sc*), showed that although an initial report was that PZM21 did not induce respiratory depression in mice breathing room air [30], this was not the case in subsequent work by others [32]. Specifically, Hill and colleagues showed that PZM21 at 40 mg/kg *sc* evoked respiratory depression in mice breathing room air with peak effects observed at 10–15 min post-dosing. Additionally, when the mice were exposed to a hypercapnic gas mixture (5% CO_2_ in air), PZM21 (10–40 mg/kg *ip*) evoked dose-dependent, naloxone-sensitive, respiratory depression characterized by reduced minute volume, but not respiratory rate, and these effects were sustained over a 60-min post-dosing period [32]. Although twice-daily injection of morphine (10 mg/kg) or PZM21 (40 mg/kg) for 5-consecutive days induced antinociceptive tolerance, tolerance did not develop to the respiratory depressant effects of PZM21 [32]. Factors potentially contributing to these between-study differences for PZM21 include differences in the dark–light cycle, differences in dosing route and between-vendor differences for mice used in these two studies [19, 33]. Also, correlations between MOP receptor/β-arrestin2 recruitment and opioid-related adverse effects has been challenged by more recent data demanding a more critical assessment and interpretation of preclinical results [34–36]. In other work, the G-protein biassed MOP receptor agonist, oliceridine, produced less respiratory depression compared with an equi-antinociceptive dose of morphine in mice [37]. However, based on the results of two Phase 3 clinical trials [38, 39], FDA approval of intravenous oliceridine was contingent upon a black box warning on life-threatening respiratory depression [8]. Regarding the possibility that KOP receptor agonism may contribute to the respiratory stimulant effects of CYX-5, previous work by others found that KOP receptor agonists had variable responses on parameters of respiration [40]. For example, KOP receptor agonists decreased respiratory frequency and minute ventilation in pentobarbital-anesthetized rats [41]. They also decreased burst frequency in isolated rat brain-spinal cord neurons and had a minimal effect on breathing in conscious SD rats [42]. However, in other work, KOP receptor agonism in conscious turtles increased minute volume [40] and DOP receptor agonism had mixed excitatory-inhibitory actions [40]. This latter observation may be due to between-species differences as opioids such as morphine evoke CNS excitatory effects in some species and depressant effects in others [40, 43].

In summary, *icv* bolus doses of CYX-5, a G-protein biassed MOP/KOP receptor agonist and a DOP receptor antagonist, evoked moderate antinociception at the highest dose tolerated (20 nmol) in rats. At the same dose, CYX-5 did not significantly impair GI motility in rats, but it inhibited castor oil-induced diarrhoea with the latter effect aligned with impaired GI motility evoked by the positive control, *icv* morphine. By comparison, we previously showed that *icv* dosing with the EM-2 analogue, CYX-6 at 20 nmol, did not inhibit either GI motility or castor oil-induced diarrhoea in rats (Table 1) [19]. This finding was somewhat unexpected as both CYX-5 and CYX-6 are G-protein biassed ligands at MOP, DOP and KOP receptors, and both are MOP receptor agonists and DOP receptor antagonists (Table 1). However, CYX-5 is a KOP receptor agonist whereas CYX-6 is a KOP receptor antagonist, and so this difference may explain their differential effects on castor oil-induced diarrhoea in rats (Table 1). Overall, our data suggest that for CYX-5, G-protein bias per se, does not abolish constipation, at least in rats. We also found differences between *icv* CYX-5 (20 nmol) and *icv* CYX-6 on parameters of respiratory function under hypercapnic conditions in rats. Specifically, *icv* CYX-5 (20 nmol), stimulated minute ventilation and tidal volume with a reduction in respiratory frequency in contrast to *icv* CYX-6 (20 nmol) that stimulated tidal volume with no changes in minute ventilation or respiratory frequency [19]. Consistent with expectations, the positive control, *icv* morphine at 100 nmol, evoked respiratory depression under hypercapnic conditions characterized by decreased minute ventilation, respiratory frequency and tidal volume, relative to animals dosed with *icv* vehicle (Fig. 5).

## Data Availability

The datasets generated during and/or analysed during the current study are available from the corresponding author on reasonable request.

## Abbreviations

%AD: Percentage of animals with diarrhoea
%SIT: Percentage of the small intestinal transit
ANOVA: One-way analysis of variance
AUC: Area under the curve
BBB: Blood–brain barrier
CNS: Central nervous system
CSW-AUC: Cumulative stool weight-area under the curve
DOP: Delta opioid
EM-2: Endomorphin-2
GI: Gastrointestinal
icv: Intracerebroventricular
ip: Intraperitoneal
KO: Knockout
KOP: Kappa opioid
MOP: Mu opioid
MV: Minute ventilation
RF: Respiratory frequency
sc: Subcutaneous
SD: Sprague Dawley
SEM: Standard error of mean
Tv: Tidal volume
WBP: Whole body plethysmography
